# The Multidomain Metaverse Cancer Care Digital Platform: Development and Usability Study

**DOI:** 10.2196/46242

**Published:** 2023-11-30

**Authors:** Sunghak Kim, Timothy Jung, Dae Kyung Sohn, Yoon Chae, Young Ae Kim, Seung Hyun Kang, Yujin Park, Yoon Jung Chang

**Affiliations:** 1 Division of Cancer Control and Policy National Cancer Center Goyang Republic of Korea; 2 Faculty of Business and Law Manchester Metropolitan University Manchester United Kingdom; 3 Center for Colorectal Cancer National Cancer Center Goyang Republic of Korea; 4 National Cancer Survivorship Center National Cancer Center Goyang Republic of Korea; 5 Planning Division Korea Smart Healthcare Association Seoul Republic of Korea

**Keywords:** metaverse, virtual reality, cancer education, cancer care, digital health, cancer treatment, patient care, cross-sectional survey, digital health intervention

## Abstract

**Background:**

As cancer treatment methods have diversified and the importance of self-management, which lowers the dependence rate on direct hospital visits, has increased, effective cancer care education and management for health professionals and patients have become necessary. The metaverse is in the spotlight as a means of digital health that allows users to engage in cancer care education and management beyond physical constraints. However, it is difficult to find a multipurpose medical metaverse that can not only be used in the field but also complements current cancer care.

**Objective:**

This study aimed to develop an integrated metaverse cancer care platform, Dr. Meta, and examine its usability.

**Methods:**

We conducted a multicenter, cross-sectional survey between November and December 2021. A descriptive analysis was performed to examine users’ experiences with Dr. Meta. In addition, a supplementary open-ended question was used to ask users for their suggestions and improvements regarding the platform.

**Results:**

Responses from 70 Korean participants (male: n=19, 27% and female: n=51, 73%) were analyzed. More than half (n=37, 54%) of the participants were satisfied with Dr. Meta; they responded that it was an interesting and immersive platform (n=50, 72%). Less than half perceived no discomfort when using Dr. Meta (n=34, 49%) and no difficulty in wearing and operating the device (n=30, 43%). Furthermore, more than half (n=50, 72%) of the participants reported that Dr. Meta would help provide non–face-to-face and noncontact services. More than half also wanted to continue using this platform in the future (n=41, 59%) and recommended it to others (n=42, 60%).

**Conclusions:**

We developed a multidomain metaverse cancer care platform that can support both health professionals and patients in non–face-to-face cancer care. The platform was uniquely disseminated and implemented in multiple regional hospitals and showed the potential to perform successful cancer care.

## Introduction

Cancer is a major global public health concern. There were approximately 19.3 million new cancer cases and 10.0 million cancer deaths globally in 2020 [1,2]. In South Korea, there were nearly 250,000 and 80,000 of such cases, respectively, in 2019 [3]. Although this disease is closely related to significant psychosocial burdens, it can be cured through early diagnosis or effective cancer treatment [4]. Thus, effective cancer education and care, which can lead to appropriate diagnosis and treatment, are important for the successful management of the disease. The dissemination of knowledge to train health professionals has become vital, especially because the types, symptoms, and treatments of cancers have diversified [5]. In addition, as the implementation of traditional cancer care methods has become difficult owing to the spread of infectious diseases such as COVID-19, self-management and home care have become more critical [6,7]. Digital health care is emerging to rapidly diffuse knowledge and is essential for cancer care.

The rapid development of information and communication technologies has accelerated digital transformation in the health care field. The world is seeking to overcome hostile environments such as the crisis of a superaged society, the outbreak of new infectious diseases, and the disparity in medical resources through digital health care [8-10]. Several studies have revealed the effectiveness of broadly applying digital health care services, including mobile health, telehealth using wearable devices, big data analytics, and artificial intelligence, to prevent and treat various diseases and maintain health [11,12].

As the digital health care industry based on innovative and evolving computing technology is expanding, metaverse technologies (eg, virtual reality [VR], augmented reality [AR], mixed reality [MR], and extended reality [XR]) that make the virtual and real worlds converge and interact are drawing attention as playing a key role in the future of digital health care [13-15]. VR is a technology that allows people to experience the real-like world in a computer-generated virtual world. AR creates an interactive environment by overlapping virtual 2D or 3D objects with the real world. In VR, users are blocked from the real world and are fully immersed in a virtual environment. However, in AR, users can simultaneously interact with virtual objects in the real world [13,16]. MR is a technology that merges real- and virtual-world elements and creates a concurrent virtuality continuum by combining the 2 worlds. In AR, virtual objects are superimposed on the real world and provide additional information such that users can see the natural environment, in which the virtual objects necessary to deliver information are placed. In MR, however, the real and virtual worlds are synchronized so that users can interact with targets in both the physical and virtual components of this shared environment [13,16]. This higher level of linkage boosts the advantages of VR and AR, making users feel that virtual components are physically present in the real world. XR is a comprehensive technology that encompasses a wide range of immersive and interactive technologies including VR, AR, and MR [13,16].

In the metaverse, people can interact with each other and digital objects in real time through avatars and engage in diverse activities, such as education, entertainment, counseling, and socializing. Metaverse provides a more immersive and interactive experience than traditional online services. In metaverse situations, users can move around and interact with digital objects, creating a sense of presence that is not possible in text-based chats or video calls [17,18]. In addition, metaverse services offer more creativity than traditional online services. In this metaverse, users have greater freedom to express themselves creatively through their avatars, build their own virtual environments, and design a wide range of virtual objects, which is not possible in a video call or traditional online game [17,18]. In addition, metaverses provide a more life-like social experience based on a more engaging way of interacting with each other than traditional online services. In this metaverse, users can explore virtual spaces, attend events, and interact with others in a way that feels more natural and authentic, which is not possible through video calls or traditional social network services [17,18]. Therefore, metaverse services can be considered to be superior to traditional online services. However, choosing a metaverse does not necessarily mean completely excluding traditional online services. A metaverse can integrate different types of online services such as videoconferencing, social media, and gaming into a single virtual space. Consequently, users can have seamless and cohesive online experiences across multiple metaverse platforms [17,18]. Although traditional online services are valuable tools for many users, through a new and transformative platform, they offer unique benefits that are not available through existing digital technologies. Previous studies have argued that metaverse technology can successfully meet the demand for non–face-to-face medical services and improve health care quality [16,19-24].

As part of efforts to scrutinize the reasons for using metaverse technology and its effectiveness, research has been conducted to identify the factors associated with the use of metaverse. Many previous studies have applied the technology acceptance model (TAM), well-known as a theoretical framework for predicting the use of new information technologies, to understand the use of metaverse better. The TAM posits that an individual’s perceived usefulness and perceived ease of use of technology determine intention to use, which consequently impacts actual use [25]. This model has been expanded by including new external and contextual factors and different factors from other theories to enhance its explanatory power in various contexts [26]. The findings of existing studies have confirmed that TAM-based factors such as perceived usefulness, perceived ease of use, and intention to use are closely associated with the use of metaverse [27-29]. In addition, according to other prior studies examining metaverse use, perceived enjoyment [30] and perceived immersion [31], whether directly or indirectly, may positively influence intention to use, while cybersickness [32] may negatively impact intention to use. Several metaverse studies have also demonstrated a positive relationship between satisfaction and intention to use [33,34]. To sum up, perceived usefulness, perceived ease of use, intention to use, perceived enjoyment, perceived immersion, and satisfaction are likely associated with using metaverse. Our research focuses on these potential variables in developing the metaverse platform and investigating its user experience.

Furthermore, the effectiveness of metaverse technology has been discussed in the context of cancer. VR, AR, and MR benefit health professionals when applied to develop and use educational or surgical tools [35,36]. Other research has shown that using VR and artificial intelligence enhances doctor-patient risk communication and shared decision-making in surgery [37]. Additionally, VR increases the satisfaction of patients with cancer under oncology care [38], improves therapy training knowledge [39], and reduces cancer-related symptoms (eg, anxiety, distress, and fatigue) [40,41]. Previous studies proposed the potential of AR in patient education and health literacy [42-44]. The multifaceted effectiveness of metaverse technology in different cancer-related groups implies that it may be useful for both cancer prevention and treatment.

Considering that every patient is different, and cancer care is complicated [45], developing and using an integrated metaverse service that can accomplish various aims are advantageous for meeting the needs of health professionals, patients, and caregivers as much as possible. Metaverse and cancer care research are in their early stages, and attempts to apply this emerging technology to cancer care are currently underway. An increasing number of studies [40,46] have demonstrated that metaverse technologies are promising in cancer care. However, these studies are not yet abundant and have been conducted on a small scale, often on single-purpose platforms. It is necessary to deal with a multipurpose platform not only to meet various needs associated with cancer care but also to understand the impact of metaverse on cancer care. To the best of our knowledge, no embodied integrated metaverse cancer care platform that embraces multiple purposes has been developed with combined functions and used in diverse real-world contexts. The challenges of the metaverse, such as inadequate funding, the necessity of careful effort to secure privacy, and an insufficient degree of portraying reality [47-49], may hinder large-scale continuous research. However, it is difficult to explain the authentic potential of metaverse technologies in cancer care simply by listing successful cases on a single platform.

Thus, this paper presents the initial phase of designing and creating a multipurpose metaverse cancer care platform that can be used and maintained steadily in the real world and investigates its usability. As this is the first study to use this new metaverse platform, there are several obstacles (eg, trial and error in developing and using the new metaverse platform, the need for training in platform management, and expensive initial setting cost) in securing many participants. Therefore, the usability test of this new platform is conducted in the form of a basic-level pilot test. Although the usability test recruits a smaller number of participants compared to previous studies and is at a basic level, mentioning the outcomes of the usability test along with the platform development information notably demonstrates the potential of the platform. In addition, despite these limitations, this study has its significance for disseminating and implementing the newly developed multidomain metaverse platform to multiple sites through a national-level project, thus laying the groundwork for large-scale research. We propose that metaverse technology may be well suited to building successful cancer care services. We expect the findings of this study to provide future directions for advancing integrated cancer care platforms.

## Methods

### Study Design

After demonstrating our cancer care platform to the users, we conducted a multicenter cross-sectional survey to examine their experiences with it.

### Procedure

#### Metaverse Platform Development (Dr. Meta)

Dr. Meta, a metaverse technology-based digital platform for cancer care, was developed at the National Cancer Center (NCC), Korea, in October 2021. It is an XR platform that combines VR and AR elements. The main purpose of its development was to overcome physical constraints (eg, time and space) and educate health professionals (eg, doctors, nurses, and pharmacists) with additional engagement and support in providing good-quality health care services to patients. The metaverse platform development project was supported by the Ministry of Science and ICT of Korea in May 2021 to foster domestic digital health care technology and promote local, non–face-to-face digital content.

Dr. Meta was developed by considering 4 types of metaverses (virtual worlds, mirror worlds, AR, and lifelogging) and their characteristics, as suggested by the Accelerated Studies Foundation [50]. First, virtual worlds are defined as alternative worlds that are similar to or completely different from the reality created by digital data. Users can access avatars whose characteristics are projected, interact with other avatars or virtual objects, and become immersed in virtual space [17,50]. This metaverse platform offers a new virtual space that is unavailable in the real world. It allows users to create their own avatars, select virtual stages or backgrounds, perform activities, and interact with virtual objects (such as 3D organs, medical devices, animals, and plants).

Second, mirror worlds are informationally enhanced worlds created by reproducing the real world. Virtual mapping, modeling, geospatial and other sensors, and lifelogging (history recording) technologies have been used to reflect real-world information as realistically as possible [17,50]. This metaverse platform contains several virtual backgrounds developed by replicating actual conference rooms at NCC Korea. This also includes real-time voice communication. These components may play a role in reflecting real-world environments.

Third, AR offers an interactive world created by overlapping virtual 2D or 3D objects in real space. Location-aware systems and networked information have been used to overlay virtual images with pictures or backgrounds of reality recognized by humans [17,50]. In this metaverse platform, users can upload, arrange, and display virtual objects (both 2D and 3D), actual files (eg, presentations, video clips, and images), and other resources. This exhibiting function may lead to an advanced combination of the real and virtual worlds.

Finally, lifelogging materializes in the digital world by recording, storing, and sharing everyday experiences and information about people and objects. Capturing memory, observation, communication, and behavior modeling with augmentation technologies are used to handle ordinary information [17,50]. This metaverse platform can provide patient charts and computed tomography scan images and has access to web services, including YouTube, Instagram, and other social network services in the virtual space. Users can upload photos and interact with others through voice conversation. They can assess their real-world experiences and information without time and spatial constraints and share them with others. Overall, the characteristics of all 4 types of metaverse were incorporated into the metaverse platform.

**Figure 1:**
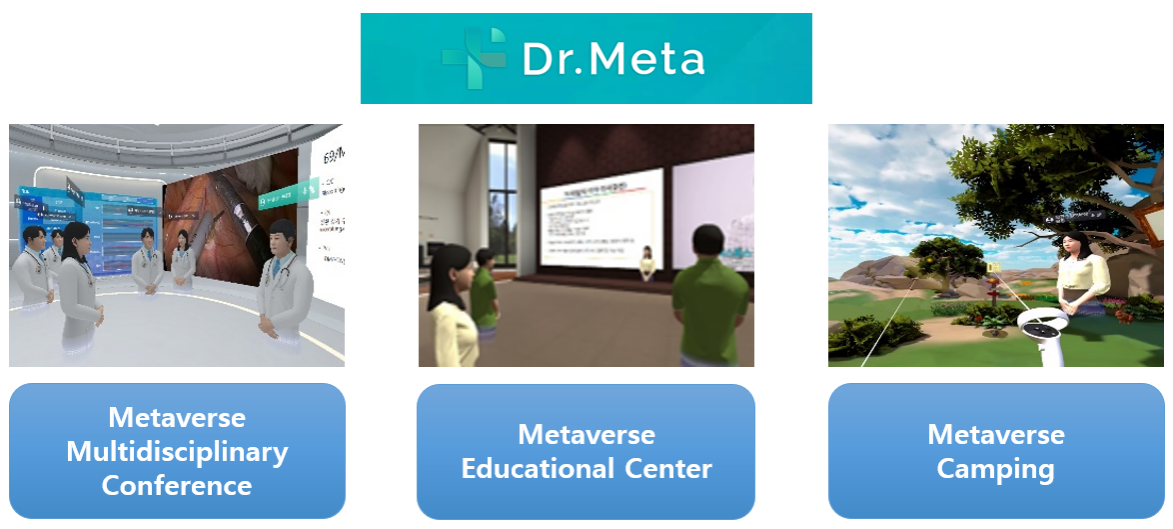
Pictures of each subplatform of Dr. Meta.

Several targeted subplatforms have been developed and operated within large platforms to achieve multidimensional goals. The metaverse platform consists of three subplatforms: (1) Metaverse Multidisciplinary Conference, (2) Metaverse Educational Center, and (3) Metaverse Camping. Metaverse Multidisciplinary Conference allows multiple health professionals from various medical fields and regions to remotely cooperate in real time by accessing the virtual space as avatars and using the 3D medical object data, patient information (eg, electronic medical record), and audio-visual data linked to the program. In Metaverse Educational Center, health professionals from other regions and medical institutions can access the virtual space of this subplatform as avatars to gain theoretical education and experience interactive medical training content. In Metaverse Camping, patients and their family members can use the XR technology in the virtual space with their relatives and acquaintances who live afar to hang out and experience psychological comfort and social support. Figure 1 shows the images of each subplatform of the metaverse platform.

As each subplatform has different purposes and targets, its characteristics and functions will inevitably be differentiated to optimize goal achievement. The Metaverse Multidisciplinary Conference and Metaverse Educational Center were developed to assist health professionals in efficient cooperation in real time and aid remote medical training for multiple people. The former tends to be more sensitive to privacy protection and security than the latter. The former focuses on patient treatment and is more likely to handle a patient’s personal health records and private information, whereas the latter focuses on knowledge instruction and skill delivery to the audience and is less likely to request sensitive personal data. Nevertheless, health professionals primarily operate these 2 subplatforms. Fundamental activities include sharing information, communicating with other users, and interacting with digital objects. Group-level performance is primarily conducted for collaboration and education, and multiple data formats are used to achieve efficient outcomes. The characteristics of all 4 types of metaverses were found on both subplatforms.

Metaverse Camping was developed to help patients and caregivers feel comfortable and supported and is mostly operated by them, even if doctors join and communicate through it. As feelings of comfort and support occur at the individual level, the functions that focus on individual-level performance or achievement predominantly comprise Metaverse Camping. There is a plan to upgrade this subplatform considering the comfort and support that can be obtained at the social level; nevertheless, at this stage, the emphasis is on individual units. Users can interact with virtual objects and perform actions such as drawing or turning on music in a virtual campsite. Virtual-world characteristics were detected on this subplatform.

After developing the metaverse platform, we disseminated it to 6 (ie, Chonnam National University Hwasun Hospital, Gyeongsang National University Hospital, Jeju National University Hospital, Kangwon National University Hospital, Kyungpook National University Chilgok Hospital, and Pusan National University Hospital) out of 12 regional cancer centers (RCCs). We also support the arrangement of the relevant infrastructure and staff training for its installation and management.

#### Study Participants

**Figure 2:**
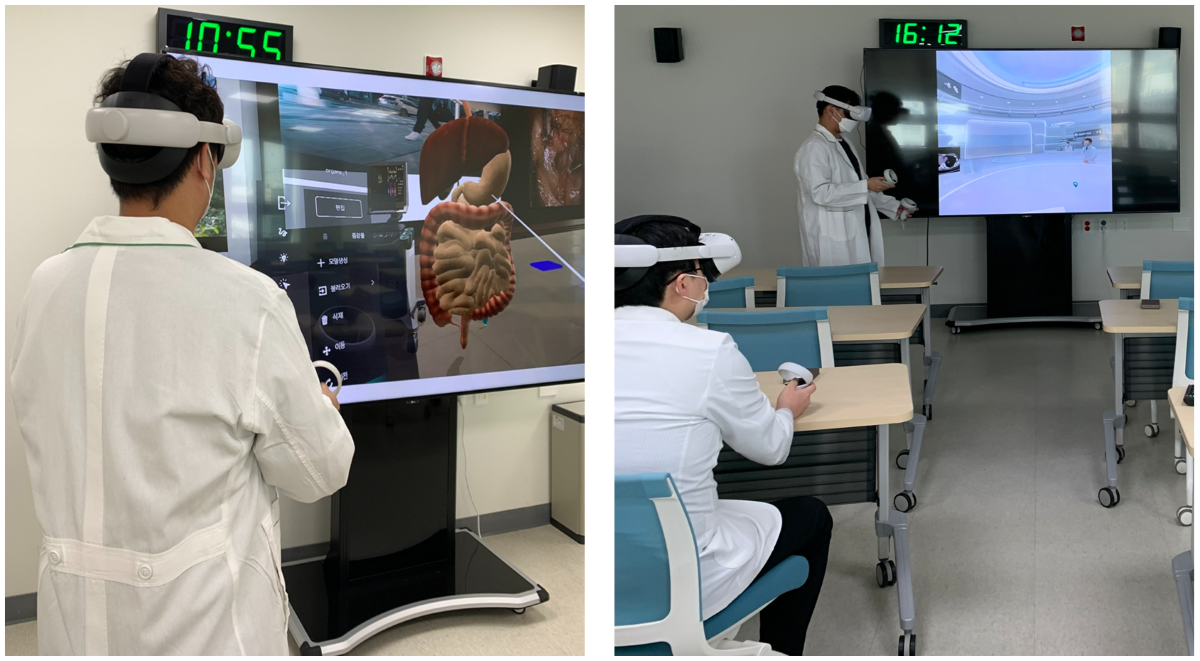
Pictures of participants using Dr. Meta.

After preparing Dr. Meta’s operations, platform demonstrations and usability tests were conducted at 7 cancer centers. Participants aged greater than 19 years were recruited from each cancer center between November and December 2021. Before participating in the survey, participants were requested to read the research information and provide their consent. They could participate in the survey only if they consented voluntarily to do so. Participant inclusion criteria for the study were being aged greater than 19 years and having experience using 1 of 3 subplatforms of this metaverse platform. Participant exclusion criteria for the study were having experience using this metaverse platform but being aged less than or equal to 19 years, being unable to complete self-reported surveys, having a serious physical or mental condition that prevents them from participating in the study, not consenting to the survey, or withdrawing consent during the study. A daily necessity worth about $5 was provided as compensation for participating in the study. We did not ask for information that could identify participants (eg, name and social security number), so the data used in this study was deidentified. Figure 2 shows the participants who used the metaverse platform.

### Measures

During the survey preparation, we asked for feedback about the initially prepared questionnaire from the platform users. We received their opinions expressing a desire to avoid feeling burdened when participating in the survey after experiencing new metaverse technology. Therefore, we used a simple and concise questionnaire to ensure that participants from various groups, including patients, could easily understand it. Researchers could universally use this questionnaire to evaluate participants’ experience using the metaverse platform across different subplatforms. The questionnaire first asked about participants’ sociodemographic information (eg, age, sex, and position in the hospital, including patients and caregivers) to assess their characteristics. The next part of the questionnaire consisted of multiple questions evaluating participants’ user experience of the metaverse platform. The questions were adapted from previous studies examining potential variables associated with using the metaverse (eg, TAM-based factors) [29,30,34], translated into Korean, abbreviated, and modified to suit the purpose and context of the study. These questions were categorized into individual differences (2 items: “I am usually interested in using new technologies or devices” and “I tend to not experience dizziness or motion sickness easily”), user satisfaction (4 items: “I was generally satisfied with using this XR platform,” “This XR platform made me feel interested and immersed,” “There was no discomfort (eg, dizziness and nausea) in using this XR platform,” and “There was no difficulty in wearing and operating this XR platform device (ie, head-mounted display and controllers)”), and future expectations (3 items: “This XR platform will be helpful for non-face-to-face and noncontact services,” “I want to continue using this XR platform in the future,” and “I want to recommend this XR platform to others”) relevant to the use of Dr. Meta. Each of them was scored on a 5-point Likert scale (1=“strongly disagree” to 5=“strongly agree”). The final part of the questionnaire consisted of an open-ended question assessing the participants’ subjective opinions (ie, suggestions and improvements) about the platform. We used these 9 items and 1 open-ended question to evaluate participants’ experiences with the metaverse platform. The questionnaire used in this study is shown in Multimedia Appendix 1.

### Statistical Analysis

We conducted a descriptive analysis to examine how participants perceived their experiences using Dr. Meta. This user experience was rated by obtaining the frequency and proportion of participants with positive opinions for each item. The number of participants with positive opinions after using the metaverse platform was calculated by adding those who answered “agree” and “strongly agree.” SPSS (version 22.0; IBM Corp) was used for the data analysis.

### Ethics Approval

This study was approved by the institutional review board of the NCC Korea and the 6 RCCs (IRB NCC: NCC2021-0331; Chonnam National University Hwasun Hospital: CNUHH-2021-218; Gyeongsang National University Hospital: GNUH2021-11-012-001; Jeju National University Hospital: JEJUNUH2021-10-017; Kangwon National University Hospital: KNUH-A-2021-11-014-001; Kyungpook National University Chilgok Hospital: KNUCH2021-11-032-001; and Pusan National University Hospital: 2111-016-109).

## Results

### Participant Characteristics

Overall, 74 people participated in the survey after using the metaverse platform, and this study analyzed the data from 70 participants. Of the 70 participants, 3 (4%), 19 (27%), 29 (41%), 15 (21%), and 4 (6%) were aged 20-29, 30-39, 40-49, 50-59, and 60-69 years, respectively. In addition, 19 (27%) participants were male, and 51 (73%) were female. More than half (n=45, 64%) of the participants were health professionals. Table 1 presents the participants’ demographic information.

**Table 1:** Demographic information of the participants.

Characteristics		Total (N=70), n (%)^a^	Metaverse Multidisciplinary Conference (n=15), n (%)	Metaverse Educational Center (n=35), n (%)	Metaverse Camping (n=20), n (%)
**Age group (years)**					
	20-29	3 (4)	0 (0)	2 (6)	1 (5)
	30-39	19 (27)	3 (20)	11 (31)	5 (25)
	40-49	29 (41)	7 (47)	15 (43)	7 (35)
	50-59	15 (21)	4 (27)	7 (20)	4 (20)
	60-69	4 (6)	1 (7)	0 (0)	3 (15)
**Sex**					
	Male	19 (27)	9 (60)	6 (17)	4 (20)
	Female	51 (73)	6 (40)	29 (83)	16 (80)
**Position in the hospital**					
	Health professionals^b^	45 (64)	14 (93)	29 (83)	2 (10)
	Patients	8 (11)	0 (0)	0 (0)	8 (40)
	Caregivers	9 (13)	0 (0)	0 (0)	9 (45)
	Others^c^	8 (11)	1 (7)	6 (17)	1 (5)

^a^Percentages may not add up to 100% due to rounding.

^b^“Health professionals” includes doctors, nurses, and pharmacists in this study.

^c^“Others” includes hospital administrative staff, educators, social workers, and researchers in this study.

### Usability Results

Regarding user satisfaction, this study’s results showed that more than half (37/70, 54%) of the participants were satisfied with using Dr. Meta. In total, 50 (72%) participants responded that Dr. Meta is an interesting and immersive platform*.* Regarding future expectations, over half (n=50, 72%) of the participants reported that the platform would be helpful for non–face-to-face and noncontact services. Furthermore, 41 (59%) participants wanted to continue using the platform in the future, and 42 (60%) wanted to recommend it to others. Table 2 summarizes the overall positive experiences of the metaverse platform’s participants.

Moreover, regarding the feedback about the metaverse platform, participants responded to the open-ended question “If you have any suggestions or improvements, please write them in.” The most commonly reported one was the difficulty in wearing and operating the XR devices (eg, “The device was heavier than I thought, and wearing it was inconvenient. It was also challenging to operate the platform”; “The device is heavy; thus, it strains my neck and shoulders”; and “People wearing glasses are uncomfortable wearing the device”). Many participants also proposed that it is crucial to improve the quality of content and create realistic materials on various topics to decrease dizziness and increase immersion (eg, “If the virtual space is more realistic, I will be more immersed.”). In contrast, the usefulness of metaverse platform in medical education was a representative advantage that the participants mentioned (eg, “It will be very helpful to the community medical field.”). Textbox 1 presents more information about the feedback on the participants’ experiences with the metaverse platform.

**Table 2:** The positive experience of the participants after using Dr. Meta.

Items^a^		Total (N=70), n (%)^b^	Metaverse Multidisciplinary Conference (n=15), n (%)	Metaverse Educational Center (n=35), n (%)	Metaverse Camping (n=20), n (%)
**Individual differences**					
	I am usually interested in using new technologies or devices.	43 (62)	13 (87)	20 (57)	10 (50)
	I tend to not experience dizziness or motion sickness easily.	24 (34)	3 (20)	11 (31)	10 (50)
**User satisfaction**					
	I was generally satisfied with using this XR^c^ platform.	37 (54)	10 (67)	19 (54)	8 (40)
	This XR platform made me feel interested and immersed.	50 (72)	13 (87)	26 (74)	11 (55)
	There was no discomfort (eg, dizziness and nausea) in using this XR platform.	34 (49)	7 (47)	16 (46)	11 (55)
	There was no difficulty in wearing and operating this XR platform device (ie, head-mounted display and controllers).	30 (43)	4 (27)	15 (43)	11 (55)
**Future expectations**					
	This XR platform will be helpful for non-face-to-face and noncontact services.	50 (72)	11 (73)	23 (66)	16 (80)
	I want to continue using this XR platform in the future.	41 (59)	11 (73)	17 (49)	13 (65)
	I want to recommend this XR platform to others.	42 (60)	11 (73)	18 (51)	13 (65)

^a^Items are based on a 5-point scale (1=“strongly disagree” to 5=“strongly agree”). The number of participants who had positive opinions after using Dr. Meta was calculated by adding the number of participants who answered “agree” and “strongly agree.”

^b^Percentages may not add up to 100% due to rounding.

^c^XR: extended reality.

**The details of feedback on the participants’ experience of Dr. Meta.**

**Advantages**

- “It will be very helpful to the community medical field.”
- “It was amazing, and I am glad my parent liked it.”
- “There was nothing particularly bad about it, and seeing virtual objects in front of me was nice. Also, it was nice to experience healing for a while.”
- “It could be useful if the convenience of using the device is improved.”
- “Depending on how you use it, it could be helpful.”
- “It was great to experience an exciting and immersive new educational system.”
- “It is livelier and engaging than face-to-face or previous online training. If the content is well-developed, it can be very effective.”
- “If improvements are made to induce interest and overcome physical constraints, this program will be a great way to deliver non-face-to-face education.”

**Improvements**

- “The device was heavier than I thought, and wearing it was inconvenient. It was also challenging to operate the platform.”
- “The device is heavy; thus, it strains my neck and shoulders.”
- “People wearing glasses feel uncomfortable wearing the device.”
- “If the virtual space is more realistic, I will be more immersed.”
- “It would be nice to have a variety of content to experience. I was getting bored with the simplicity over time.”
- “It was hard to use, and the voice function needed to be fixed.”
- “It was inconvenient to wear and use the device. Adding a function that allows users to experience content without the device would be wonderful.”
- “I need some guidance on how to operate it.”
- “The device is heavy and requires time to get used to the operation.”
- “The content should be designed considering different age groups and health conditions.”
- “I am worried about contamination problems when multiple people share the device.”
- “I wonder if applying this platform in the medical field is realistic because of the expensive cost of the device.”

## Discussion

### Principal Findings

This study aimed to (1) develop an integrated metaverse cancer care platform and (2) examine its user experience. Dr. Meta is a multipurpose metaverse health care platform distributed and used by the local community. To the best of our knowledge, it is not only the first to be developed in South Korea but is also a unique case worldwide. This was possible because this study used a cancer center system established at the national level. Our results showed that more than half of users thought this metaverse platform was satisfying, interesting, and immersive. Although less than half of users indicated positive opinions on no discomfort in using this platform and no difficulty in wearing and operating its device, more than half of users thought it was helpful for non–face-to-face and noncontact services and wanted to use it continuously and recommend it to others. These outcomes were also observed in the pattern of responses to a feedback question. Users primarily mentioned the difficulty in wearing and operating this platform’s device. They also raised the necessity of improving the quality of its content. On the other hand, users were favorable about the platform’s usefulness as a medical education tool. The metaverse platform will serve as evidence to enable the expansion of the country’s medical metaverse project, helping to establish a concrete direction for future research and development. It will also assist in overcoming physical constraints and proceeding with successful non–face-to-face cancer care, positively contributing to solving unequal issues of medical resource accessibility between areas (urban, metro, and rural regions).

Moreover, the findings of this study showed that various groups can use this metaverse platform according to their requirements. When it comes to the subplatforms targeting health professionals, more than half of users thought Metaverse Multidisciplinary Conference and Metaverse Educational Center were satisfying, interesting, and immersive. Even though less than half of users provided positive opinions on no discomfort in using these subplatforms and no difficulty in wearing and operating their device, more than half of users thought these subplatforms were helpful for non–face-to-face and noncontact services and wanted to recommend them to others. Over half of the users wanted to use Metaverse Multidisciplinary Conference continuously but not Metaverse Educational Center. Although the initial cost for the development of metaverse platform was relatively high, health professionals could learn repeatedly at no additional cost once the platform was deployed. Based on the results of other studies using a medical metaverse for health professionals’ education [51-53], it is feasible to predict that this platform will be more effective in providing education that cannot be easily implemented in the real world. It can also serve as a catalyst to activate the training of health professionals by enabling education on invasive treatment [54]. To maximize these advantages, a capability that the virtual world can accurately reflect the real world is needed [55,56]. This metaverse platform can be developed in the future to embody digital twins for this purpose. This should be accomplished through discussions on data sharing and privacy issues.

With respect to the subplatform targeting patients and caregivers, while less than half of users thought Metaverse Camping was satisfying, more than half of users thought it was interesting and immersive. Unlike the other 2 subplatforms, this subplatform received positive opinions on no discomfort in using it and no difficulty in wearing and operating its device from over half of users. In addition, more than half of users thought it was helpful for non–face-to-face and noncontact services and wanted to use it continuously and recommend it to others. Through this subplatform, patients can overcome physical constraints and receive support such as high-quality cancer education, monitoring, and counseling. Consequently, they can enhance self-efficacy regarding health behaviors and gain comfort, similar to other studies [57-59] examining the role of the medical metaverse in patient support. A more immersive environment may provide a greater patient experience without being disturbed by external stimuli [55,60-62]. This metaverse platform can be developed in the future to establish an immersive environment for this purpose. Additional solutions that maximize immersion should be developed, such as preparing more accessories to overcome the inconvenience of wearing such devices.

Furthermore, Dr. Meta provided evidence of a metaverse platform for conducting more sophisticated research. It can act as a stimulus in new studies that test the associations among relevant variables. Verifying the determinants or constructing predictive models using this metaverse platform could be directions for future research. For example, examining the role of immersion in the satisfaction and continuous use of metaverse platform could be an important research topic. These outcomes can guide the theory-driven development of other metaverse health care platforms for different disease contexts.

### Future Perspective

The study results prove that metaverse platform has the potential to provide effective cancer care and simultaneously show that it needs to be upgraded to enhance the user experience. Some interesting differences among the subplatforms suggest that certain components of metaverse platform require further improvement. This study’s outcomes showed that less than half of the Metaverse Educational Center users wanted to continuously use the Metaverse Educational Center. In addition, less than half of Metaverse Camping users responded that they were satisfied with using Metaverse Camping. We plan to set different future directions to improve subplatforms by accommodating their own purposes.

Regarding Metaverse Educational Center, an interactive environment where users can directly control and interact with virtual objects and spatial elements may allow them to become more engaged with the platform [60-62]. Such improved engagement may help users achieve their goal of sharing information successfully. For example, diverse interactive functions such as performing physical activities through motion recognition, assembling and disassembling 3D anatomical structures, and manipulating 3D medical devices may be useful for delivering messages and acquiring knowledge. These improvements are expected to be widely applicable in other health care fields. In addition to high interactivity, vivid 2D or 3D digital objects that match topics or innovative simulation functions that realistically describe topics may also strengthen users’ intentions toward continuous use [63].

Concerning Metaverse Camping, gamification elements (eg, quizzes and rewards) and personalized feedback may help users successfully attain the goal of self-motivation and feeling comfortable [64,65]. This may enhance user satisfaction with the platform. For instance, if users can customize their characters, navigate campsites, complete quests, receive fruit stickers, or watch flower images appearing on trees as rewards, they may experience comfort, fun, or other positive emotions. Thus, users can dynamically interact with new virtual objects and easily immerse themselves in the platform. Among the 4 types of metaverse, these plans are closely related to virtual worlds.

Moreover, improving the voice, movement, and time spent recording functions on both subplatforms may elicit a more positive user experience. Communication within a shorter time interval in the Metaverse Educational Center or recoding the voice diary on Metaverse Camping may make users think that they are putting their daily life information on the platform and that it is tailored. As people tend to favor personalized information [66], these upgrades may encourage them to use the platform. Among the 4 metaverse types, these plans are closely related to lifelogging. In summary, we plan Dr. Meta’s future development by focusing more on the virtual world and lifelogging aspects. It is expected that the influence of these improvements will not only be within the subplatform but will also positively affect the evaluation and actual use of the entire platform by stimulating user satisfaction and curiosity.

Furthermore, it is possible to present meaningful directions for future development and maintenance beyond the scope of subplatform improvement. Currently, Dr. Meta is mainly used through the head-mounted display (HMD); however, many participants complained of motion sickness due to the HMD or said that it was difficult to wear and handle. Therefore, we plan to develop different versions of the metaverse platform that can be operated on various media types, such as desktops and tablets, to reduce the dependence on HMD. We will also develop a user-friendly manual to guide the use of Dr. Meta* *and provide it to the users to minimize inconvenience. In addition, according to their feedback, several participants felt content needed improvement. For example, they stated that the content was monotonous and lacked realism. Thus, we will emphasize vivid and realistic depictions of content that need to be authentic and fantasy elements of content that need to be fictional. We also plan to increase the number of content scenarios and videos that users can choose. Additionally, some participants suggested that providing content that reflects user characteristics might enhance the quality of services. Therefore, in the future, we will prepare various content and matching algorithm systems to satisfy user requirements. Finally, current subplatforms and content are still insufficient to cover the complexity of cancer care. Therefore, we will gradually expand the metaverse platform by developing new subplatforms tailored to target participant groups to diversify its purposes and functions. While securing various subplatforms, we will spur Dr. Meta’s* *operation by applying models and content well-fitted with topics.

While advancing Dr. Meta, we sought to diversify its uses. Our research is currently supported by the Ministry of Health and Welfare of Korea starting in 2022, linked to a government project that focuses on establishing infrastructure and maintaining its setting to disseminate and implement new digital health care technologies in local communities. We aimed to establish an environment in which we could cooperate with health professionals from other hospitals in multiple regions of South Korea. In addition, a platform will be developed to collaborate on education or research with Koreans as well as foreign hospitals and research institutes.

### Limitations

Although there were favorable evaluations and expectations of this platform, this study had some limitations. First, the sample size seems to be small. As this was the first study conducted following the development of metaverse platform, the main focus was to explore the field applicability of the newly developed metaverse platform called Dr. Meta. Therefore, although there was a limit to securing a sufficient number of users, this study first examined the feasibility of metaverse platform by conducting a usability test in the form of a pilot test on a small number of people to provide evidence of the potential for practical use of the platform. To address this issue, we will invite new RCCs to our project and recruit participants for an extended period in future research. Dr. Meta is expected to operate more stably in the future; therefore, more participants are expected to be recruited.

Second, it looks like the proportion of participants is unbalanced by position in the hospital. The number of participants who were patients or caregivers was small, and health professionals mainly participated in the study. It is a natural occurrence because 2 of the 3 subplatforms introduced in this study were developed for health professionals, and we even wanted to test the subplatform developed for patients on health professionals first before providing it to patients to ensure safety and appropriateness. Still, it is important to have an even distribution of sufficient participants for good-quality data analysis. We will consider performing large-scale participant recruitment along with applying a statistical methodology that deals with the percentage of participants of each subgroup in the population or its representation, such as stratified sampling, to collect adequate data inclusively in future research.

Third, although presenting the metaverse platform’s development information and its outcomes of usability test together shows that the platform is promising for successful cancer care, the basic level of this usability test seems difficult to deal with more elaborate research questions. For example, there was an absence of use of existing validated questionnaires in this study. This study was conducted as an early-stage pilot study, and a questionnaire developed for an exploratory purpose was used rather than a structured one. Furthermore, this study aimed to include patients among the overall participants, so we needed to consider their understanding of the questionnaire. It was decided to use a theory-based but straightforward, easy, and short questionnaire because patients typically need care and can easily get tired after experiencing new metaverse technologies and platforms. It provided flexibility and ease of conducting usability assessments in the real-world field. On the other hand, it was limited in its ability to scrutinize the relationships between variables related to user satisfaction. As this was the first study to use the metaverse platform, it was inevitable that this study focused on introducing its development in detail. Nonetheless, to verify this multidomain platform’s potential, we undertook its usability test together regardless of the test level. Considering that medical metaverse research is currently in its infancy, this study may encourage scholars to go beyond the medical metaverse research centered on reviews or single-domain platform studies and practitioners to adopt a large-scale medical metaverse platform. We will design future studies that intensively investigate a target subplatform’s features and content based on sufficient participants and an advanced understanding of the topic. For a sophisticated examination of the metaverse platform’s user experience and influence, we will upgrade our current questionnaire by adding more theory-based simple items asking about potential other new variables related to the usability of the medical metaverse. We will also consider using a systematically validated usability questionnaire when planning a future study examining a target subplatform with a fixed participant group and topic.

### Conclusions

To the best of our knowledge, this study is the first to verify the possibility of developing multidomain metaverse cancer care platforms in South Korea that have the potential for successful non–face-to-face cancer care. This study was the starting point for examining the effectiveness of this metaverse cancer care platform. We will develop the metaverse platform to strengthen user immersion and tailor experiences by highlighting virtual worlds and lifelogging. If the development of hardware devices and software content suitable for this purpose is combined, this medical metaverse platform will successfully ease the burden on patients with cancer. Moreover, the platform has been disseminated and implemented for multiple RCCs. Future developments may solve physical distance and disparities in access to medical resources more effectively. The metaverse platform will be an alternative cancer education method for health professionals against COVID-19 and will be especially helpful for patients not living in urban areas to obtain cancer care benefits. This research provides not only the development of simple medical metaverse platform functions but also an environmental model that can facilitate the sustainable use of the platform. We will progress in more extended future research with more participants to enrich evidence about the effectiveness of Dr. Meta* *and discover more in-depth scientific insight about this multidomain metaverse cancer care platform.

## Appendix

Multimedia Appendix 1 The questionnaire used to evaluate participants' experience using the metaverse platform.

## Abbreviations

AR: augmented reality
HMD: head-mounted display
MR: mixed reality
NCC: National Cancer Center
RCC: regional cancer center
TAM: technology acceptance model
VR: virtual reality
XR: extended reality

## Data Availability

The data sets generated or analyzed during this study are available from the corresponding author on reasonable request.
