# Supplementary material for: The Multidomain Metaverse Cancer Care Digital Platform: Development and Usability Study
Source: JMIR Serious Games. 2023 Nov 30;11:e46242. doi: 10.2196/46242 (PMC10722376; doi:10.2196/46242)
Supplement: Multimedia Appendix 1 [file games_v11i1e46242_app1.pdf]

Metaverse Platform User Experience Test

We are investigating Dr. Meta's (the metaverse platform) user experience to provide a better quality of platform environment and services to those who have used the platform. Please give us your honest evaluation and opinions on the platform.

1. Consent to Participate in the Program & Consent to Collection and Use of Personal Information

1) I agree to participate in the program voluntarily. ☐ I agree ☐ I do NOT agree

2) I agree to the collection and use of my personal information. ☐ I agree ☐ I do NOT agree

2. Participation Information

1) Organization: ________________ 2) Date of Use: ________________

3) Sub-Platform:

☐ Metaverse Multidisciplinary Conference ☐ Metaverse Educational Center

☐ Metaverse Camping

3. Basic Information

1) Sex: ☐ Male ☐ Female 2) Age: ______________

3) Position in the Hospital:

☐ Health Professionals (Doctors, Nurses, Pharmacists, etc.) ☐ Patients

☐ Caregivers ☐ Others (Hospital Administrative Staffs, Educators, Social Workers, Researchers, etc.)

4. Questions are asking about your thoughts about Dr. Meta after using it. Please indicate how much you agree with the statement below.

#	Items	Response				
		Strongly Disagree	Disagree	Neutral	Agree	Strongly Agree
Individual differences						
1	I am usually interested in using new technologies or devices.	①	②	③	④	⑤
2	I tend to not experience dizziness or motion sickness easily.	①	②	③	④	⑤
User satisfaction						
3	I was generally satisfied with using this XR platform.	①	②	③	④	⑤
4	This XR platform made me feel interested and immersed.	①	②	③	④	⑤
5	There was no discomfort (eg, dizziness and nausea) in using this XR platform.	①	②	③	④	⑤
6	There was no difficulty in wearing and operating this XR platform device (ie, head-mounted display and controllers).	①	②	③	④	⑤
Future expectations						

7	This XR platform will be helpful for non-face-to-face and noncontact services.	①	②	③	④	⑤
8	I want to continue using this XR platform in the future.	①	②	③	④	⑤
9	I want to recommend this XR platform to others.	①	②	③	④	⑤

5. If you have any suggestions or improvements, please write them in.

Thank you for participating in this survey.
